# Comparison of Maternal and Neonatal Antibody Levels After COVID-19 Vaccination vs SARS-CoV-2 Infection

**DOI:** 10.1001/jamanetworkopen.2022.40993

**Published:** 2022-11-09

**Authors:** Dustin D. Flannery, Sigrid Gouma, Miren B. Dhudasia, Sagori Mukhopadhyay, Madeline R. Pfeifer, Emily C. Woodford, Sara M. Briker, Jourdan E. Triebwasser, Jeffrey S. Gerber, Jeffrey S. Morris, Madison E. Weirick, Christopher M. McAllister, Scott E. Hensley, Karen M. Puopolo

**Affiliations:** 1 Division of Neonatology, Children’s Hospital of Philadelphia, Philadelphia, Pennsylvania; 2 Department of Pediatrics, University of Pennsylvania Perelman School of Medicine, Philadelphia; 3 Clinical Futures, Children’s Hospital of Philadelphia, Philadelphia, Pennsylvania; 4 Department of Microbiology, University of Pennsylvania Perelman School of Medicine, Philadelphia; 5 Department of Obstetrics and Gynecology, University of Michigan, Ann Arbor; 6 Division of Infectious Diseases, Children’s Hospital of Philadelphia, Philadelphia, Pennsylvania; 7 Department of Biostatistics, Epidemiology and Informatics, University of Pennsylvania Perelman School of Medicine, Philadelphia

## Abstract

**Importance:**

Pregnant persons are at an increased risk of severe COVID-19 from SARS-CoV-2 infection, and COVID-19 vaccination is currently recommended during pregnancy.

**Objective:**

To ascertain the association of vaccine type, time from vaccination, gestational age at delivery, and pregnancy complications with placental transfer of antibodies to SARS-CoV-2.

**Design, Setting, and Participants:**

This cohort study was conducted in Pennsylvania Hospital in Philadelphia, Pennsylvania, and included births at the study site between August 9, 2020, and April 25, 2021. Maternal and cord blood serum samples were available for antibody level measurements for maternal-neonatal dyads.

**Exposures:**

SARS-CoV-2 infection vs COVID-19 vaccination.

**Main Outcomes and Measures:**

IgG antibodies to the receptor-binding domain of the SARS-CoV-2 spike protein were measured by quantitative enzyme-linked immunosorbent assay. Antibody concentrations and transplacental transfer ratios were measured after SARS-CoV-2 infection or receipt of COVID-19 vaccines.

**Results:**

A total of 585 maternal-newborn dyads (median [IQR] maternal age, 31 [26-35] years; median [IQR] gestational age, 39 [38-40] weeks) with maternal IgG antibodies to SARS-CoV-2 detected at the time of delivery were included. IgG was detected in cord blood from 557 of 585 newborns (95.2%). Among 169 vaccinated persons without SARS-CoV-2 infection, the interval from first dose of vaccine to delivery ranged from 12 to 122 days. The geometric mean IgG level among 169 vaccine recipients was significantly higher than that measured in 408 persons after infection (33.88 [95% CI, 27.64-41.53] arbitrary U/mL vs 2.80 [95% CI, 2.50-3.13] arbitrary U/mL). Geometric mean IgG levels were higher after vaccination with the mRNA-1273 (Moderna) vaccine compared with the BNT162b2 (Pfizer/BioNTech) vaccine (53.74 [95% CI, 40.49-71.33] arbitrary U/mL vs 25.45 [95% CI, 19.17-33.79] arbitrary U/mL; *P* < .001). Placental transfer ratios were lower after vaccination compared with after infection (0.80 [95% CI, 0.68-0.93] vs 1.06 [95% CI, 0.98-1.14]; *P* < .001) but were similar between the mRNA vaccines (mRNA-1273: 0.70 [95% CI, 0.55-0.90]; BNT162b2: 0.85 [95% CI, 0.69-1.06]; *P* = .25). Time from infection or vaccination to delivery was associated with transfer ratio in models that included gestational age at delivery and maternal hypertensive disorders, diabetes, and obesity. Placental antibody transfer was detectable as early as 26 weeks’ gestation. Transfer ratio that was higher than 1.0 was present for 48 of 51 (94.1%) births at 36 weeks’ gestation or later by 8 weeks after vaccination.

**Conclusions and Relevance:**

This study found that maternal and cord blood IgG antibody levels were higher after COVID-19 vaccination compared with after SARS-CoV-2 infection, with slightly lower placental transfer ratios after vaccination than after infection. The findings suggest that time from infection or vaccination to delivery was the most important factor in transfer efficiency.

## Key Points

**Question:**

Is placental antibody transfer after COVID-19 vaccination different from that after SARS-CoV-2 infection in pregnant individuals?

**Findings:**

In this cohort study of 585 maternal-newborn dyads, maternal and cord blood IgG antibody levels were higher after vaccination compared with after infection. An association was observed between time from infection or vaccination to delivery and transfer ratio.

**Meaning:**

Findings of this study suggest that time from infection or vaccination to delivery was the most important factor in transfer ratio efficiency.

## Teaser

This cohort study examines the response to COVID-19 vaccination, exposure to SARS-CoV-2 infection, timing of infection or vaccination, and placental antibody transfers among pregnant persons and their newborns.

## Introduction

Pregnant persons are at an increased risk of severe COVID-19 caused by SARS-CoV-2 infection. Pregnancy is associated with an increased risk of mechanical ventilation, intensive care unit admission, and death from COVID-19.^1^ COVID-19 during pregnancy may also be a factor in increased risk of stillbirth and complications, such as preeclampsia, preterm birth, and neonatal intensive care unit admission.^2,3^ Although newborns appear to be at a lower risk of severe COVID-19, there are reports of serious neonatal infection and attributable death, and infants are at risk of hospitalization related to SARS-CoV-2 infection.^4,5,6,7,8^

COVID-19 vaccines became available in the US in December 2020. Pregnant persons were excluded from early clinical trials, resulting in uncertainty regarding vaccine administration during pregnancy.^4^ With evolving evidence demonstrating both vaccine safety in pregnancy and increased risk of severe infection during pregnancy, the Centers for Disease Control and Prevention released an urgent health advisory in September 2021 that strongly recommended COVID-19 vaccination for pregnant persons.^9^ Recent evidence suggests that COVID-19 vaccines are immunogenic and effective in pregnant persons and that maternally derived antibodies can be transferred across the placenta to the newborn after vaccination during pregnancy, as observed after SARS-CoV-2 infection.^10,11,12,13,14,15,16,17,18^ Most studies to date have been limited by small numbers of vaccinated persons, qualitative antibody assays, exposure to a single vaccine type, or self-report of vaccination.

In the midst of the global COVID-19 pandemic, vaccination at any time with any available vaccine is recommended to acutely protect pregnant persons from the disease. As the COVID-19 pandemic evolves, optimal use of available vaccines will be informed by comparative data on vaccine response among pregnant persons and by detailed understanding of vaccination timing to ensure maximal placental antibody transfer. In this study, we leveraged a large cohort of maternal and cord blood serum samples that were tested for antibodies to SARS-CoV-2. The objective was to ascertain the association of vaccine type, time from vaccination, gestational age at delivery, and pregnancy complications with placental transfer of antibodies to SARS-CoV-2.

## Methods

### Study Setting and Population

This cohort study was conducted at Pennsylvania Hospital in Philadelphia, Pennsylvania. Pregnant persons who gave birth at the study site between August 9, 2020, and April 25, 2021, and their newborns were included. The institutional review board of the University of Pennsylvania approved this study and waived the informed consent requirement because the study posed minimal risk and could not practicably be performed without waiver of consent. We followed the Strengthening the Reporting of Observational Studies in Epidemiology (STROBE) reporting guideline.

### Data Collection

Demographic and clinical data, including timing of SARS-CoV-2 exposures and symptoms, SARS-CoV-2 nasopharyngeal polymerase chain reaction (PCR) test results, and vaccine type (messenger RNA [mRNA] vaccine BNT162b2 [Pfizer/BioNTech] and mRNA-1273 [Moderna]; adenovirus vector vaccine Ad26.COV2.S [Johnson & Johnson]) and dates of administration, were collected from review of electronic medical records. Only the first neonates from multiple-gestation deliveries were included in all analyses. Maternal-newborn dyads with incomplete medical records were excluded. Race and ethnicity were self-reported on hospital admission; these data were included given the known disparities in SARS-CoV-2 infection and COVID-19 vaccination. Prepregnancy body mass index (calculated as weight in kilograms divided by height in meters squared) from the first prenatal visit was abstracted from the medical record or from the patient’s self-reported entry in birth registration. *International Statistical Classification of Diseases and Related Health Problems, Tenth Revision* diagnosis codes were validated and used to identify hypertensive disorders and diabetes, as previously described.^19^

Preterm delivery was defined as less than 37 weeks’ gestation, and term delivery was defined as 37 weeks’ gestation or later. During the study period, pregnant persons were routinely screened for SARS-CoV-2 using nasopharyngeal PCR testing when admitted to the hospital for childbirth; testing could also be performed before the pregnancy or earlier during pregnancy and outside of the health system. Persons with medical record report of SARS-CoV-2 symptoms and confirmatory positive result from nasopharyngeal PCR testing were considered to have a symptomatic infection. Symptomatic illness was defined according to definitions provided by the National Institutes of Health.^20^ Persons with antibodies to SARS-CoV-2 but without a record of symptomatic illness or COVID-19 vaccination were considered to have asymptomatic infection whether or not they had a positive nasopharyngeal PCR test result.

### Serum Collection and Testing

Pregnant persons routinely have blood drawn for rapid plasma reagin testing when admitted to the hospital for childbirth, and cord blood is routinely collected for newborn blood type and direct antiglobulin testing. Collection of residual serum samples from these specimens was performed as previously described.^19,21^ Serum samples were fully deidentified before antibody level measurements; when results were available, persons who were seropositive were reidentified for manual medical record review by one of us (K.M.P.). The IgG and IgM antibodies to the receptor-binding domain of the SARS-CoV-2 spike protein were measured using enzyme-linked immunosorbent assay; this quantitative assay has been previously described.^19^ Serum samples with IgG and/or IgM concentrations of more than 0.48 arbitrary U/mL were considered to be seropositive; concentrations below this cutoff were considered to be seronegative and were assigned a value of 0.24 arbitrary U/mL for statistical analysis.

### Statistical Analysis

The demographic, clinical, and antibody characteristics of pregnant persons and newborns were compared according to the following 3 exposures: (1) asymptomatic infection, (2) symptomatic infection, and (3) COVID-19 vaccination with or without infection. Transfer ratios were calculated as newborn IgG concentration divided by maternal IgG concentration. Antibody concentrations and transfer ratios were reported as geometric mean concentrations with 95% CIs and were log_2_-transformed for statistical analyses. We used scatter diagrams and Spearman rank correlation coefficients to assess the associations between transfer ratio and duration from onset of symptoms or the first positive PCR test result among persons with symptomatic infection or between first vaccine dose and delivery. Mann-Whitney test was used to compare the duration from onset of symptoms or the first positive PCR test result or first vaccine dose to delivery, χ^2^ test was used to compare seropositivity, and an unpaired, 2-tailed *t* test was used to compare antibody concentrations and transfer ratios between the analytic groups. We constructed linear regression models to explore the associations between log_2_-transformed transfer ratio and time from infection or vaccination to delivery, gestational age at delivery, and maternal factors (hypertensive disorders, diabetes, and obesity) that may change placental function.

Two-sided *P* < .05 was considered to be statistically significant. Stata, version 16 (StataCorp LLC) and Prism, version 9 (GraphPad) were used for statistical analyses.

## Results

### Study Population

**Figure 1.**
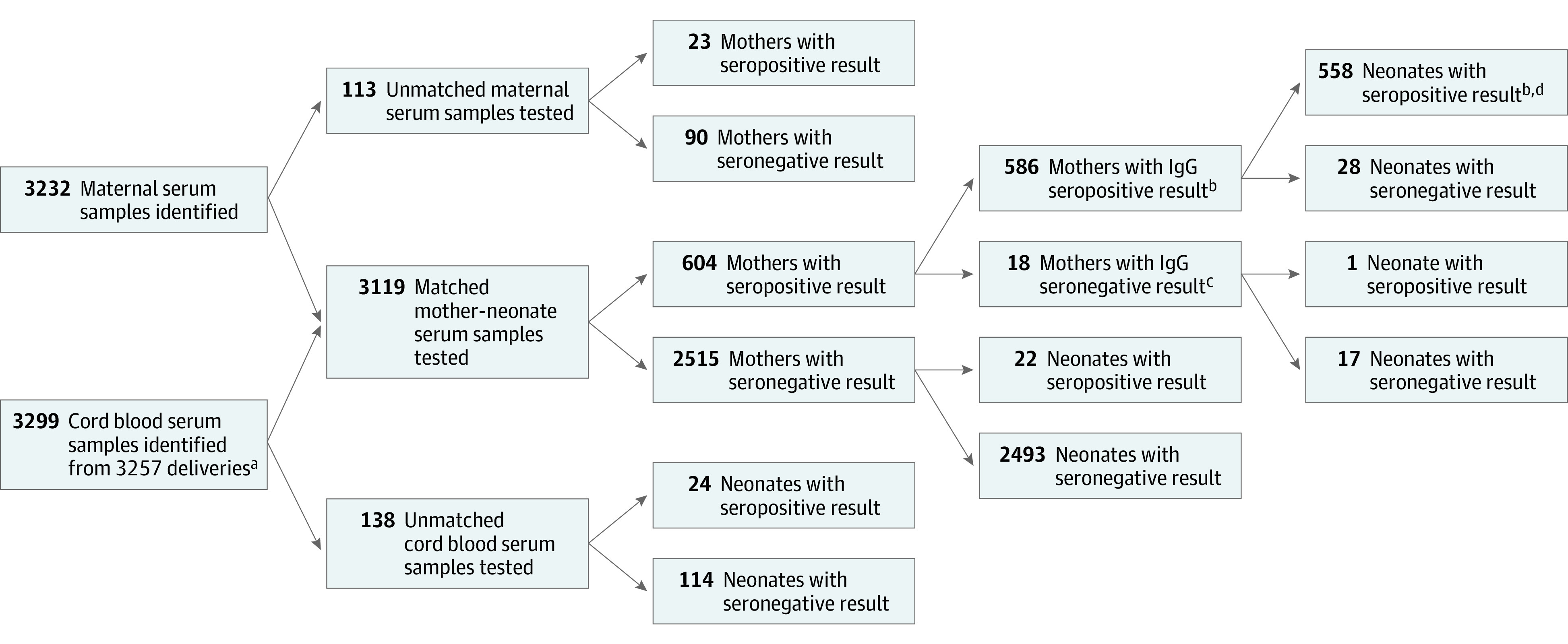
Participant Flow Diagram ^a^Included 47 twin deliveries. ^b^One maternal-newborn dyad was excluded from analysis due to incomplete medical records. ^c^Mothers were seropositive only for IgM antibodies to SARS-CoV-2. ^d^Included only the first twin from 2 sets of twins. Seropositivity in unmatched maternal or cord blood serum samples was not different from that in matched samples.

The study cohort consisted of 585 maternal-newborn dyads, with childbirth occurring at a median (IQR) maternal age of 31 (26-35) years and at a median (IQR) gestational age of 39 (38-40) weeks; 31 neonates (5.3%) were born at less than 37 weeks’ gestation (Table 1). The cohort was derived from the 3381 pregnant persons who gave birth during the study period, among whom matched maternal and cord blood serum samples were available for 3119 maternal-newborn dyads (92.3%) (Figure 1). Antibodies to SARS-CoV-2 were detected in 604 pregnant persons (19.4%): 18 (3.0%) had IgM only, 380 (62.9%) had IgG only, and 206 (34.1%) had both IgG and IgM. Because IgM was not expected to cross the placenta, further analyses were restricted to the 585 dyads with maternal IgG and available data (Figure 1). Among these 585 dyads, IgG was detected in cord blood from 557 newborns (95.2%). Of the 28 persons who were seropositive with newborns who were seronegative, the geometric mean IgG concentrations were lower compared with the geometric mean IgG concentrations of persons paired with newborns who were seropositive (1.21 [95% CI, 0.90-1.61] arbitrary U/mL vs 6.46 [95% CI, 5.63-7.42] arbitrary U/mL; *P* < .001).

**Table 1.** Demographic Characteristics of Study Cohort

Characteristic	Patients, No. (%)						
	All patients (n = 585)	SARS-CoV-2 infection (n = 408)		Vaccination against SARS-CoV-2 (n = 177)			
		Asymptomatic^a^ (n = 265)	Symptomatic (n = 143)	BNT162b2 (Pfizer/BioNTech) (n = 104)	mRNA-1273 (Moderna) (n = 60)	Ad26.COV2.S (Johnson & Johnson) (n = 2)	Unknown (n = 11)
Maternal age, median (IQR), y	31 (26-35)	28 (23-32)	31 (27-34)	34 (31-36)	34 (32-35)	33 (28-37)	33 (32-35)
Race and ethnicity^b^							
Asian	35 (6.0)	11 (4.2)	7 (4.9)	10 (9.6)	7 (11.7)	0	0
Hispanic	117 (20.0)	82 (30.9)	26 (18.2)	8 (7.7)	0	1 (50.0)	0
Non-Hispanic Black	177 (30.3)	124 (46.8)	48 (33.6)	2 (1.9)	2 (3.3)	0	1 (9.1)
Non-Hispanic White	249 (42.6)	45 (17.0)	61 (42.7)	82 (78.9)	50 (83.3)	1 (50.0)	10 (90.9)
Other^c^	7 (1.2)	3 (1.1)	1 (0.7)	2 (1.9)	1 (1.7)	0	0
Gravidity							
1	181 (30.9)	76 (28.7)	39 (27.3)	40 (38.5)	20 (33.3)	1 (50.0)	5 (45.5)
2	174 (29.7)	75 (28.3)	42 (29.4)	29 (27.9)	23 (38.3)	0	5 (45.5)
≥3	230 (39.3)	114 (43.0)	62 (43.4)	35 (33.7)	17 (28.3)	1 (50.0)	1 (9.1)
Prepregnancy BMI							
<18.0	6 (1.0)	1 (0.4)	1 (0.7)	2 (1.9)	2 (3.3)	0	0
18.0 to <25.0	243 (41.5)	91 (34.3)	45 (31.5)	67 (64.4)	32 (53.3)	1 (50.0)	7 (63.6)
25.0 to <30.0	159 (27.2)	69 (26.0)	46 (32.2)	25 (24.0)	16 (26.7)	0	3 (27.3)
≥30.0	171 (29.2)	101 (38.1)	49 (34.3)	9 (8.7)	10 (16.7)	1 (50.0)	1 (9.1)
Missing data	6 (1.0)	3 (1.1)	2 (1.4)	1 (1.0)	0	0	0
Hypertension	151 (25.8)	71 (26.8)	34 (23.8)	25 (24.0)	16 (26.7)	0	5 (45.5)
Diabetes	54 (9.2)	21 (7.9)	16 (11.2)	9 (8.7)	7 (11.7)	1 (50.0)	0
GA at delivery, median (IQR), wk	39 (38-40)	39 (38-40)	39 (38-39)	39 (38-40)	39 (38-39)	39.5 (39-40)	39 (39-40)
Preterm, GA<37 wk	31 (5.3)	14 (5.3)	9 (6.3)	4 (3.9)	4 (6.7)	0	0
Birth weight, median (IQR), g	3280 (2950-3610)	3260 (2930-3590)	3260 (2960-3600)	3290 (3045-3575)	3380 (2830-3670)	3710 (3620-3800)	3330 (3070-3820)
Newborn male sex	291 (49.7)	131 (49.4)	73 (51.1)	54 (51.9)	26 (43.3)	1 (50.0)	6 (54.6)
Newborn female sex	294 (50.3)	134 (50.6)	70 (48.9)	50 (48.1)	34 (56.7)	1 (50.0)	5 (45.4)

Abbreviations: BMI, body mass index (calculated as weight in kilograms divided by height in meters squared); GA, gestational age.

^a^ Included 4 pregnant persons who declined testing.

^b^ Race and ethnicity were self-reported.

^c^ Included 1 individual who self-identified as mixed race, 3 who identified as other race and ethnicity, and 2 for whom race and ethnicity data were missing. One individual declined to answer.

Demographic characteristics of the study cohort are shown in Table 1. Patients self-identified as being of Asian (35 [6.0%]), Hispanic (117 [20.0%]), non-Hispanic Black (177 [30.3%]), non-Hispanic White (249 [42.6%]), or other (7 [1.2%], including mixed, other, and unknown) race and ethnicity. Of the 585 dyads, 265 (45.3%) had asymptomatic infection and 143 (24.4%) had symptomatic infection. At least 1 dose of a COVID-19 vaccine was administered before delivery in 177 pregnant persons (30.3%): 104 received BNT162b2, 60 received mRNA-1273, and 2 received Ad26.COV2.S. In 11 cases (6.2%), vaccine type was not recorded. A second dose of vaccine was administered before delivery to 126 of 164 persons (76.8%) who were known to be vaccinated with an mRNA vaccine. Compared with individuals with SARS-CoV-2 infection, those vaccinated were older, more often of non-Hispanic White race and ethnicity, and more often had prepregnancy body mass index higher than 30.

### Antibody Concentration

Antibody concentrations associated with SARS-CoV-2 infection and COVID-19 vaccination are shown in Table 2. To compare the response to infection vs vaccination, we excluded from this analysis 8 vaccinated persons with a history of infection. The IgG concentrations were higher among persons with symptomatic compared with asymptomatic infection (Table 2). The median (IQR) duration between first vaccine dose and delivery was 42 (26-63) days and was not different between recipients of the BNT162b2 and mRNA-1273 vaccines (Table 2). Two doses of vaccine were administered before delivery to 79% of BNT162b2 vaccine recipients and 77% of mRNA-1273 vaccine recipients. As shown in Table 2, maternal and cord IgG concentrations were higher among mRNA-1273 vaccine recipients compared with BNT162b2 vaccine recipients.

**Table 2.** Antibody Levels and Transfer Ratios Associated With SARS-CoV-2 Infection and COVID-19 Vaccination

	Infection				Vaccination^a^						*P* value^b^
	Geometric, mean (95% CI)				Geometric, mean (95% CI)						
	All patients (n = 408)	Asymptomatic (n = 265)^c^	Symptomatic (n = 143)	*P* value	All patients (n = 169)	BNT162b2 (Pfizer/BioNTech) (n = 97)	mRNA-1273 (Moderna) (n = 60)	Ad26.COV2.S (Johnson & Johnson) (n = 2)	Unknown (n = 10)	*P* value^d^	
Time from infection or vaccination to delivery, median (IQR), d^e^	77 (27-141)	1 (0-15)	97 (49-149)	<.001	42 (26-63)	41 (25-61)	43 (30-65)	26 (23-28)	62 (57-66)	.28	<.001
Maternal IgG concentration, arbitrary U/mL	2.80 (2.50-3.13)	2.41 (2.10-2.75)	3.71 (3.05-4.51)	<.001	33.88 (27.64-41.53)	25.45 (19.17-33.79)	53.74 (40.49-71.33)	7.04 (0.17-283.21)	46.72 (19.04-114.65)	<.001	<.001
Cord blood IgG concentration >0.48, arbitrary U/mL, No. (%)	382 (93.6)	248 (93.6)	134 (93.7)	.96	167 (98.8)	96 (99.0)	59 (98.3)	2 (100.0)	10 (100.0)	>.99	.006
Cord blood IgG concentration, arbitrary U/mL^f^	2.97 (2.63-3.35)	2.55 (2.21-2.95)	3.94 (3.19-4.87)	<.001	27.05 (21.04-34.78)	21.66 (15.19-30.89)	37.76 (26.03-54.79)	2.94 (0.00-6116.01)	49.25 (20.11-120.60)	.04	<.001
Transfer ratio	1.06 (0.98-1.14)	1.06 (0.96-1.17)	1.06 (0.94-1.20)	.99	0.80 (0.68-0.93)	0.85 (0.69-1.06)	0.70 (0.55-0.90)	0.42 (0.01-21.60)	1.05 (0.56-1.98)	.25	<.001

^a^ Eight vaccinated pregnant persons with known positive polymerase chain reaction test results were excluded from analysis.

^b^ *P* values represent comparison of all persons with SARS-CoV-2 infection (N = 408) vs all persons with COVID-19 vaccination (N = 169).

^c^ Included 4 pregnant persons who declined testing.

^d^ *P* values represent comparison of BNT162b2 (Pfizer/BioNTech) with mRNA-1273 (Moderna) vaccines.

^e^ Time from infection to delivery was measured as time from onset of symptoms or first positive polymerase chain reaction test result to delivery and reported for all 143 persons in the symptomatic group and 38 persons in the asymptomatic group with a positive polymerase chain reaction test result before delivery. Time from vaccination to delivery was measured as time from first dose of vaccine to delivery; date of first vaccine dose was missing for 10 persons (1 in the BNT162b2, 1 in the mRNA-1273, and 8 in the unknown group).

^f^ A total of 28 neonates (26 among persons with SARS-CoV-2 infection and 2 among vaccinated persons) were seronegative, with cord blood IgG concentration less than 0.48 arbitrary U/mL. Cord blood IgG concentration for these 28 neonates was set at 0.24 arbitrary U/mL and included in analysis.

The geometric mean maternal IgG concentration of the 169 vaccine recipients without infection was significantly higher compared with the geometric mean IgG concentration of the 408 persons with infection (33.88 [95% CI, 27.64-41.53] arbitrary U/mL vs 2.80 [95% CI, 2.50-3.13] arbitrary U/mL; *P* < .001). Similarly, the geometric mean cord blood IgG concentration of neonates born to vaccine recipients was significantly higher compared with the geometric mean cord blood IgG concentration of the neonates born to persons with infection (Table 2). A plot of maternal IgG concentration and time from symptomatic, PCR test result–confirmed, well-dated infections or first dose of vaccine to delivery (in days) shows that generally higher antibody levels were associated with vaccination compared with infection (eFigure 1 in the Supplement).

### Transfer Ratios

The geometric mean transfer ratio among persons with infection was higher than the geometric mean transfer ratio among vaccinated persons (Table 2). However, transfer ratio that was higher than 1.0 was present for 48 of 51 (94.1%) births at 36 weeks’ gestation or later by 8 weeks after vaccination. The geometric mean transfer ratio was similar between symptomatic and asymptomatic pregnant persons with infection, and between BNT162b2 and mRNA-1273 vaccine recipients (Table 2). Placental transfer ratios were lower after vaccination compared with after infection (0.80 [95% CI, 0.68-0.93] vs 1.06 [95% CI, 0.98-1.14]; *P* < .001) but were similar between the mRNA vaccines (BNT162b2: 0.85 [95% CI, 0.69-1.06]; *P* = .25; mRNA-1273: 0.70 [95% CI, 0.55-0.90]).

**Figure 2.**
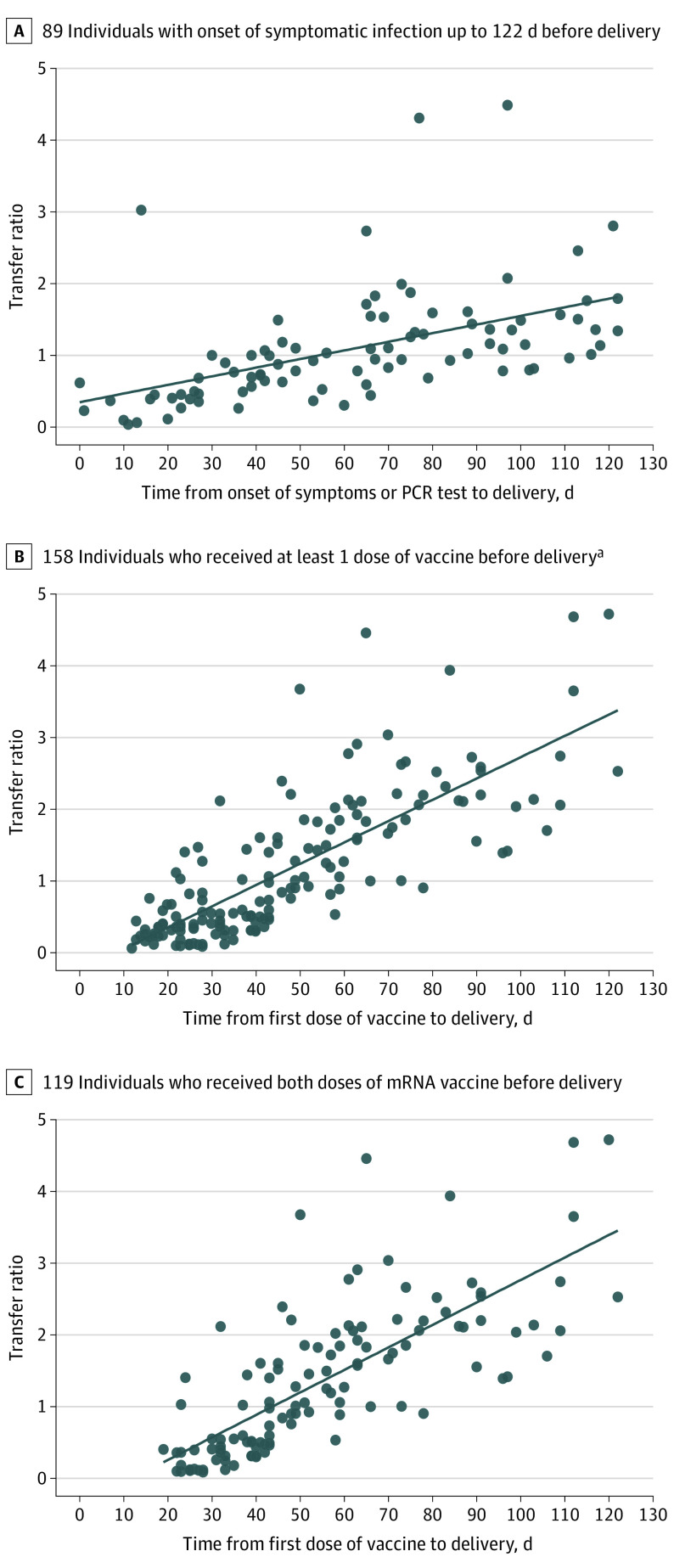
Association Between Placental Transfer Ratio and Time From SARS-CoV-2 Infection or First Vaccine Dose to Delivery There was a correlation between transfer ratio and time from symptom onset or polymerase chain reaction (PCR) testing to delivery (*r* = 0.6885; *P* < .001) (A) and between transfer ratio and time from first vaccine dose to delivery among persons who received at least 1 dose of an mRNA vaccine before delivery (*r* = 0.8126; *P* < .001) (B) and among persons who received both doses of an mRNA vaccine before delivery (*r* = 0.8137; *P* < .001) (C). ^a^One outlier value of transfer ratio greater than 15 is not shown in the figure.

The range for onset of infection to delivery among symptomatic persons was 0 to 384 days, and the first dose of vaccine to delivery among all vaccinated (but without infection) persons ranged in duration from 12 to 122 days; among vaccinated persons after 2 vaccine doses, the range was 19 to 122 days. To ascertain the association between time of infection or vaccination and transfer ratio, we plotted the transfer ratio against time (expressed as days) between onset of symptomatic infection or date of vaccination and delivery. Visual inspection of the plot of all 143 persons with onset of symptomatic infection suggested that the association was not linear over the 384-day range (eFigure 2 in the Supplement). Therefore, in a post hoc analysis, we compared 89 persons with onset of symptomatic infection up to 122 days before delivery (Figure 2A), 159 persons with known date of first dose of vaccine (Figure 2B), and 119 persons with known dates for 2 doses of vaccine (Figure 2C). In each case, the transfer ratio increased linearly over time.

**Figure 3.**
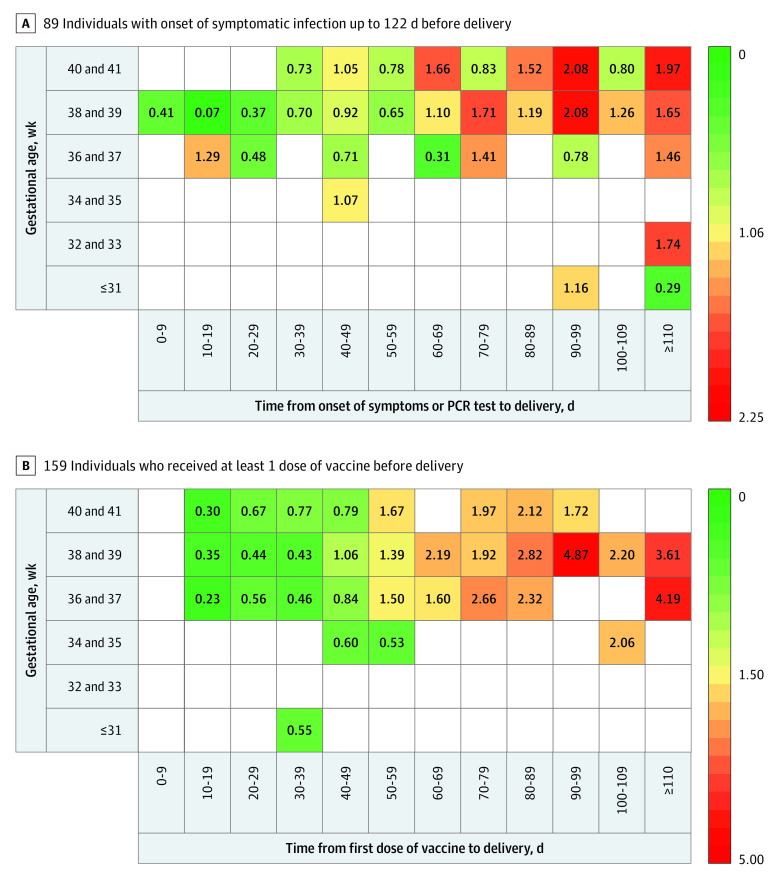
Heat Map of Mean Transfer Ratio by Gestational Age vs Time From SARS-CoV-2 Infection or First Vaccine Dose to Delivery Each box displays the mean transfer ratio for the corresponding gestational age vs time from infection or first vaccine dose to delivery category among all persons contributing data (eFigure 3 in the Supplement). PCR indicates polymerase chain reaction.

The study population included neonates born as early as 23 weeks’ gestation; 23 persons with infection and 8 persons who were vaccinated delivered at less than 37 weeks’ gestation. Placental antibody transfer was detectable as early as 26 weeks’ gestation. When comparing maternal-newborn dyads with neonates born at less than 37 weeks’ gestation or at 37 weeks’ gestation or later, no significant differences were found between geometric mean maternal and cord blood IgG concentrations after infection or vaccination or in geometric mean transfer ratio after infection or vaccination (eTable 1 in the Supplement). To further explore the association of gestational age at delivery with time from symptomatic infection or vaccination, we generated heat maps of mean transfer ratios (Figure 3) and conducted linear regression analyses. The distribution of dyads in each group in the heat map (Figure 3) is shown in eFigure 3 in the Supplement.

In linear regression models, we observed a significant association between time from infection or vaccination to delivery and transfer ratio on bivariate analysis, and these associations remained significant in models that included gestational age at delivery and maternal hypertensive disorders, diabetes, and obesity (eTable 2 in the Supplement). For example in the bivariate model, for each day increase in time from infection to delivery, transfer ratio increased by 0.02 (95% CI, 0.01-0.02; *P* < .001), and for each day increase in time from vaccination to delivery, transfer ratio increased by 0.03 (95% CI, 0.03-0.03; *P* < .001). In contrast, gestational age at delivery was not associated with transfer ratio on bivariate analysis for infection, and this association remained nonsignificant when accounting for the additional factors (eTable 2 in the Supplement).

## Discussion

Among persons who gave birth between August 9, 2020, and April 25, 2021, at Pennsylvania Hospital, IgG antibodies to the SARS-CoV-2 spike protein were present in higher concentrations after vaccination with mRNA vaccines compared with the antibody levels present after symptomatic or asymptomatic infection and were associated with higher cord blood antibody levels after maternal vaccination compared with maternal infection. Placental transfer ratios were slightly lower after maternal vaccination compared with maternal infection. An increase in transfer ratio was found both with longer duration of time between SARS-CoV-2 exposure (via infection or vaccination) and delivery and with increasing gestational age at delivery. However, multivariate modeling that accounted for the duration of time from infection or vaccination to delivery, gestational age at delivery, and maternal pregnancy comorbidities found that the time from infection or vaccination to delivery was the dominant factor associated with placental transfer. We believe this study expands on the evolving data on COVID-19 vaccination during pregnancy by using deidentified sample collection to minimize consent bias, assessing placental antibody transfer among patients with diverse demographic characteristics, and comparing response to asymptomatic and symptomatic SARS-CoV-2 infection with different COVID-19 vaccines at varying durations between first dose and delivery.

We found that antibody levels after vaccination with an mRNA vaccine were at least 10-fold higher than the levels after infection. Furthermore, antibody levels were higher after vaccination with the mRNA-1273 vaccine compared with the BNT162b2 vaccine. Previous studies of smaller numbers of pregnant persons also found that the quantitative IgG response to mRNA vaccines had higher maternal and cord blood IgG antibody levels compared with levels after infection, but these studies were unable to compare IgG responses by specific vaccine types.^22,23,24^ The reasons for higher antibody levels after the mRNA-1273 vaccine were unclear but may be associated with a higher antigen dose with the mRNA-1273 vaccine compared with the BNT162b2 vaccine. A similar proportion of persons received both doses of each mRNA vaccine and received each vaccine type at similar times before delivery. A comparative effectiveness study conducted among US veterans (92.7% of whom were men, with a median age of 67 years) also found that antibody levels were higher after vaccination with the mRNA-1273 vs the BNT162b2 vaccine.^25^ The present study did not address the effectiveness of vaccination in preventing infection among pregnant persons or newborns, although a recent study reported on the effectiveness of maternal vaccination in protecting young infants from SARS-CoV-2 infection.^26^

As a novel human exposure, SARS-CoV-2 presents an important opportunity to study placental transfer kinetics given the clear initial timing of exposure at different points during pregnancy and the lack of previous immunity in most cases. Placental transfer of antibodies that are present from conception at low levels is distinct from transfer of antibodies that are present from conception and boosted during pregnancy, such as the intent with tetanus toxoid–reduced diphtheria toxoid–acellular pertussis vaccine administration during pregnancy. In addition, IgG subclass, variation in maternal antibody levels at conception; maternal comorbidities, such as HIV infection; and placental dysfunction that may be present in cases of severe fetal growth restriction or maternal preeclampsia have been described as playing a role in transfer efficiency.^27^ Several case reports and series to date have assessed maternal antibody response to different COVID-19 vaccines at various gestations and dynamics of placental antibody transfer after vaccination.^10,15,16,17,28,29^ Beharier et al^14^ reported efficient placental antibody transfer, with transfer ratios generally higher than 1 when the first dose of a vaccine was administered more than 14 days before delivery in a cohort of 86 pregnant persons in Israel who were vaccinated with BNT162b2 . Nir et al^23^ reported a correlation between maternal serum and cord blood antibody concentrations in a study that compared 64 women who were vaccinated with the BNT162b2 vaccine with 11 parturient women who had COVID-19 during pregnancy.

We did not observe a significant difference in transfer ratio between persons with asymptomatic and those with symptomatic infection, consistent with previous initial findings.^21^ However, none of the symptomatic persons with infection were critically ill; therefore, we could not establish whether maternal critical illness is a factor in placental transfer. We explored the contributions of time from infection or vaccination to delivery and gestational age at delivery, revealing that each variable had an independent association with placental antibody transfer. We found no difference in maternal IgG level or transfer efficiency for preterm vs term deliveries when accounting for time from vaccination to delivery. Only a small portion of this cohort had documented SARS-CoV-2 infection before the current pregnancy, but transfer ratios were still robust; the longest interval between infection and delivery that we observed was 384 days, with a transfer ratio of 1.2. As expected, transfer ratios were affected by preterm delivery, although we observed transfer as early as 26 weeks’ gestation at delivery and a transfer ratio higher than 0.5 for vaccine-elicited antibodies as early as 29 weeks’ gestation at delivery. Because antibody levels after vaccination were higher than those after infection, cord blood IgG levels among the 8 cases of preterm delivery in vaccinated persons were significantly higher than the levels observed among cases of term delivery after SARS-CoV-2 infection.

### Strengths and Limitations

The strengths of this study include the large cohort of pregnant persons with infection and vaccination during the initial phases of the COVID-19 pandemic; diversity in race and ethnicity of the population; pregnancy comorbidities and gestational age at delivery; comparison of antibody responses to different vaccine types; and wide range of timing of infection and vaccination relative to delivery, which provided information on both vaccine response and transplacental antibody dynamics.

This study also has limitations. To ascertain the history of infection and vaccination status, we relied on electronic medical record review. We cannot rule out the possibility that some persons whom we deemed to be vaccinated and without infection may have had asymptomatic infections or that some persons whom we deemed to have infection and to be unvaccinated after vaccines became available may have had undocumented vaccinations. We were unable to perform assays for antibodies associated with infection (such as nucleocapsid IgG), which could definitively rule out infection among the vaccinated cohort. In this deidentified study of discarded specimens, we were unable to assess the antibody content in breastmilk, duration and durability of newborn antibody, or degree of newborn protection from infection. Furthermore, we did not identify the IgG subclass of vaccine-elicited antibodies.

## Conclusions

This cohort study found that concentrations of maternal IgG antibodies to SARS-CoV-2 in pregnant persons after vaccination with mRNA vaccines were higher than the levels after viral infection, but placental antibody transfer ratios were lower after vaccination than after infection. Placental transfer and cord blood IgG concentration were detectable as soon as 15 days after the first dose of an mRNA vaccine, and transfer ratios increased for several weeks after the first vaccine dose. These findings suggest that time from infection or vaccination to delivery was the most important factor in transfer efficiency, and these findings can inform optimal COVID-19 vaccination strategy during pregnancy.

## Notes

Supplement. **eFigure 1.** Maternal Antibody (IgG) Concentration Over Time From Infection or First Dose of Vaccine to Delivery **eFigure 2.** Relationship Between Placental Transfer Ratio and Time From Infection to Delivery Among Pregnant Persons With Symptomatic Disease **eTable 1.** SARS-CoV-2 Antibody Levels and Transfer Ratios for Term vs. Preterm Births **eFigure 3.** Number of Pregnant Persons Contributing Data to Each Gestational Age vs. Time From Infection or First Vaccine Dose to Delivery Category for its Counterpart Figure of Heat Map (Figure 3, Panels A and B) **eTable 2.** Linear Regression of Transfer Ratio vs. Gestational Age and/or Time From Infection or First Vaccine Dose to Delivery
