# Supplementary material for: Comparison of Maternal and Neonatal Antibody Levels After COVID-19 Vaccination vs SARS-CoV-2 Infection
Source: JAMA Netw Open. 2022 Nov 9;5(11):e2240993. doi: 10.1001/jamanetworkopen.2022.40993 (PMC9647482; doi:10.1001/jamanetworkopen.2022.40993)
Supplement: Supplement. — eFigure 1. Maternal Antibody (IgG) Concentration Over Time From Infection or First Dose of Vaccine to Delivery eFigure 2. Relationship Between Placental Transfer Ratio and Time From Infection to Delivery Among Pregnant Persons With Symptomatic Disease eTable 1. SARS-CoV-2 Antibody Levels and Transfer Ratios for Term vs. Preterm Births eFigure 3. Number of Pregnant Persons Contributing Data to Each Gestational Age vs. Time From Infection or First Vaccine Dose to Delivery Category for its Counterpart Figure of Heat Map (Figure 3, Panels A and B) eTable 2. Linear Regression of Transfer Ratio vs. Gestational Age and/or Time From Infection or First Vaccine Dose to Delivery [file jamanetwopen-e2240993-s001.pdf]

Supplemental Online Content

Flannery DD, Gouma S, Dhudasia MB, et al. Comparison of maternal and neonatal antibody levels after COVID-19 vaccination vs SARS-CoV-2 infection. *JAMA Netw Open*. 2022;5(11):e2240993. doi:10.1001/jamanetworkopen.2022.40993

eFigure 1. Maternal Antibody (IgG) Concentration Over Time From Infection or First Dose of Vaccine to Delivery

eFigure 2. Relationship Between Placental Transfer Ratio and Time From Infection to Delivery Among Pregnant Persons With Symptomatic Disease

eTable 1. SARS-CoV-2 Antibody Levels and Transfer Ratios for Term vs. Preterm Births

eFigure 3. Number of Pregnant Persons Contributing Data to Each Gestational Age vs. Time From Infection or First Vaccine Dose to Delivery Category for its Counterpart Figure of Heat Map (Figure 3, Panels A and B)

eTable 2. Linear Regression of Transfer Ratio vs. Gestational Age and/or Time From Infection or First Vaccine Dose to Delivery

This supplemental material has been provided by the authors to give readers additional information about their work.

© 2022 Flannery DD et al. *JAMA Network Open.*

eFigure 1. Maternal Antibody (IgG) Concentration Over Time From Infection or First Dose of Vaccine to Delivery

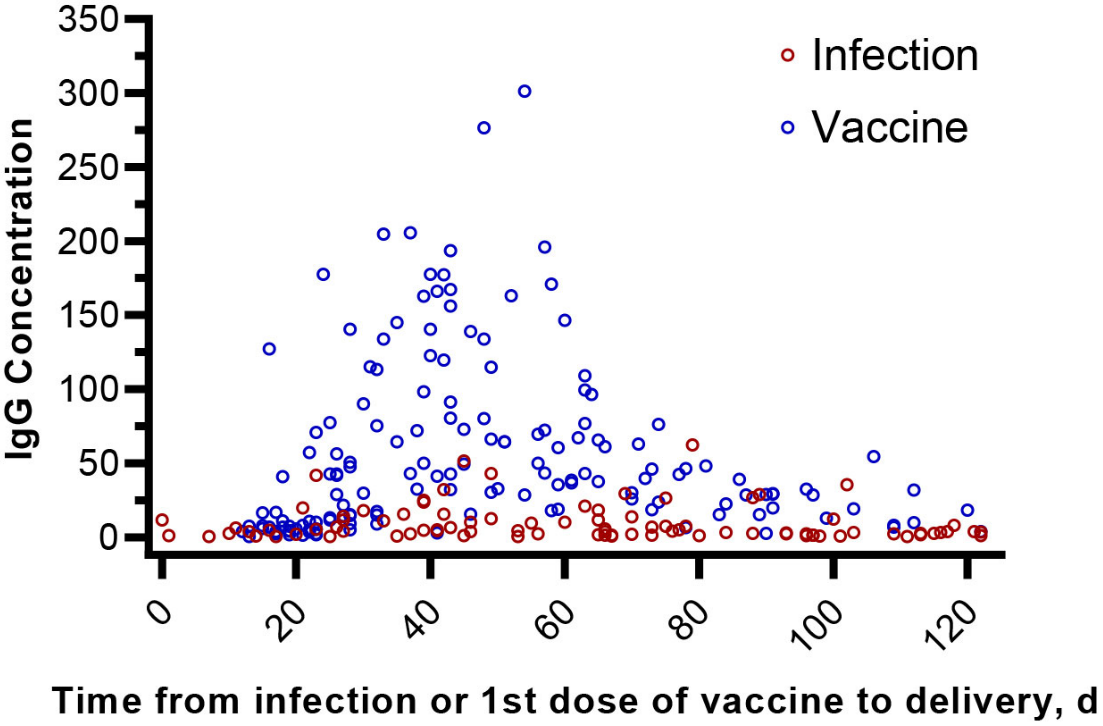

Footnote: A scatter plot comparing maternal IgG concentration over time from infection or first dose of vaccine to delivery. It includes 89 pregnant persons with onset of symptomatic SARS-CoV-2 infection up to 122 days prior to delivery (red circles) and 159 pregnant persons with SARS-CoV-2 vaccine with known date of first dose of vaccine (blue circles). Three outlier values (all in the vaccine group) of IgG concentration >500 arbitrary units/mL are not shown in the figure. *IgG*, immunoglobulin G; *SARS-CoV-2*, severe acute respiratory syndrome coronavirus 2.

© 2022 Flannery DD et al. *JAMA Network Open.*

eFigure 2. Relationship Between Placental Transfer Ratio and Time From Infection to Delivery Among Pregnant Persons With Symptomatic Disease

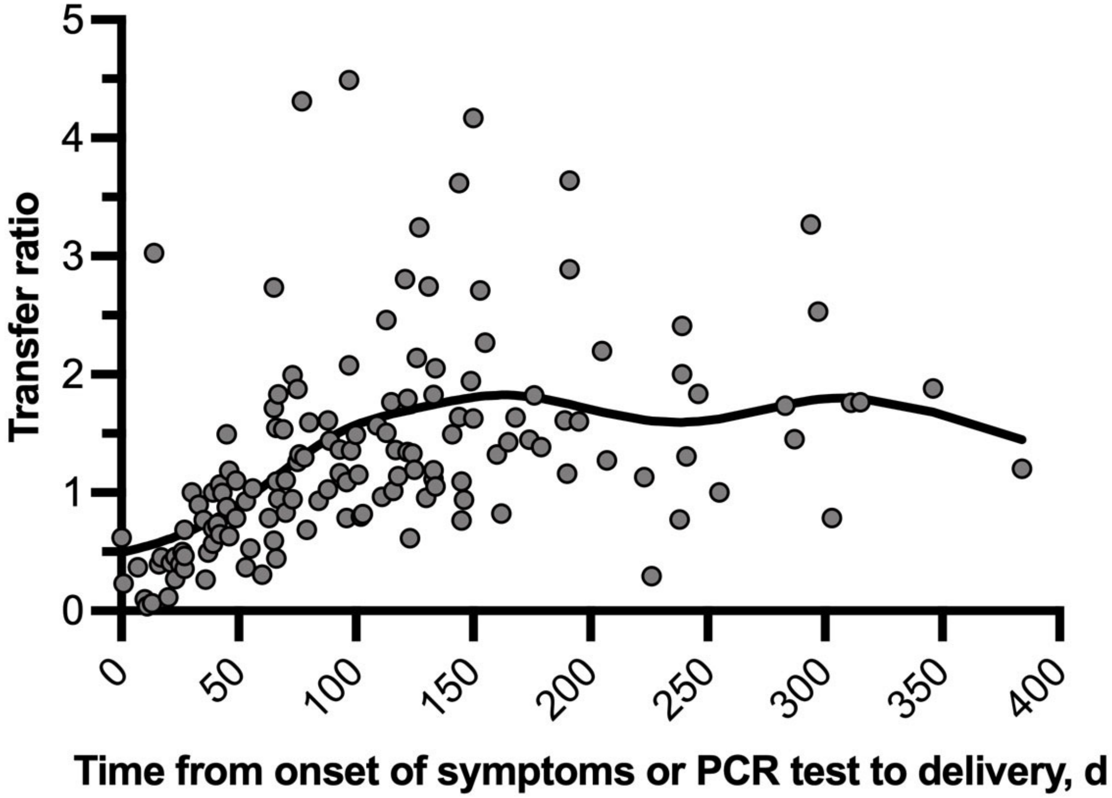

Footnote: A smooth penalized spline was fitted to this scatterplot using the “gam” package in R, with smoothing parameter selected by generalized cross validation. *PCR*, polymerase chain reaction.

© 2022 Flannery DD et al. *JAMA Network Open.*

eTable 1. SARS-CoV-2 Antibody Levels and Transfer Ratios for Term vs. Preterm Births

	**Delivery GA ≥37 weeks**	**Delivery GA <37 weeks**	***P* value**
**SARS-CoV-2 Infection**	**N = 385**	**N = 23**	
GA at delivery, median (range), wk:d	39:2 (37:0-41:5)	34:5 (23:3-36:6)	<0.001
Maternal IgG concentration, geometric mean (95% CI)	2.78 (2.47-3.12)	3.22 (2.00-5.19)	0.55
Cord IgG concentration[a], geometric mean (95% CI)	2.99 (2.64-3.39)	2.60 (1.48-4.55)	0.59
Transfer ratio[a], geometric mean (95% CI)	1.08 (1.00-1.17)	0.81 (0.61-1.06)	0.08
**SARS-CoV-2 Vaccine[b]**	**N = 161**	**N = 8**	
GA at delivery, median (range), wk:d	39:3 (37:0-41:6)	35:3 (29:6-36:6)	<0.001
Maternal IgG concentration, geometric mean (95% CI)	33.80 (27.44-41.63)	35.61 (10.53-120.40)	0.91
Cord IgG concentration[a], geometric mean (95% CI)	27.34 (21.13-35.39)	21.82 (5.30-89.91)	0.71
Transfer ratio[a], geometric mean (95% CI)	0.81 (0.69-0.95)	0.61 (0.25-1.48)	0.45

Footnote: [a]Twenty-six of 408 infants born to IgG-seropositive persons with SARS-CoV-2 infection and 2 of 169 infants born to IgG-seropositive persons with SARS-CoV-2 vaccine were seronegative (cord IgG ≤0.48 arbitrary U/mL); cord IgG concentration was set at 0.24 arbitrary U/mL for these 28 infants and included in analysis. [b]Eight vaccinated pregnant persons who were also known PCR positive were excluded from analysis. *CI*, confidence interval; *GA*, gestational age; *IgG*, immunoglobulin G; *SARS-CoV-2*, severe acute respiratory syndrome coronavirus 2.

© 2022 Flannery DD et al. *JAMA Network Open.*

eFigure 3. Number of Pregnant Persons Contributing Data to Each Gestational Age vs. Time From Infection or First Vaccine Dose to Delivery Category for its Counterpart Figure of Heat Map (Figure 3, Panels A and B)

A

Number of Contributing Data Points

Gestational age	0 to 9	10 to 19	20 to 29	30 to 39	40 to 49	50 to 59	60 to 69	70 to 79	80 to 89	90 to 99	100 to 109	≥110
40 and 41	0	0	0	2	3	2	4	1	2	1	1	3
38 and 39	3	3	6	6	5	2	6	7	3	4	4	6
36 and 37	0	3	3	0	2	0	1	2	0	1	0	1
34 and 35	0	0	0	0	1	0	0	0	0	0	0	0
32 and 33	0	0	0	0	0	0	0	0	0	0	0	0
≤31	0	0	0	0	0	0	0	0	0	1	0	0

Time from onset of symptoms or PCR test to delivery, d

B

Number of Contributing Data Points

Gestational age	0 to 9	10 to 19	20 to 29	30 to 39	40 to 49	50 to 59	60 to 69	70 to 79	80 to 89	90 to 99	100 to 109	≥110
40 and 41	0	9	6	7	4	8	0	2	1	3	0	0
38 and 39	0	7	20	12	15	7	11	8	4	5	3	2
36 and 37	0	3	5	2	5	1	1	1	1	0	0	2
34 and 35	0	0	0	0	1	1	0	0	0	0	1	0
32 and 33	0	0	0	0	0	0	0	0	0	0	0	0
≤31	0	0	0	1	0	0	0	0	0	0	0	0

Time from 1st dose of vaccine to delivery, d

Footnote: *PCR*, polymerase chain reaction.

© 2022 Flannery DD et al. *JAMA Network Open.*

eTable 2. Linear Regression of Transfer Ratio vs. Gestational Age and/or Time From Infection or First Vaccine Dose to Delivery

Cohort	Variable	Model 1			Model 2			Model 3	
		Change in transfer ratio (95% CI)	*P* value		Change in transfer ratio (95% CI)	*P* value		Change in transfer ratio (95% CI)	*P* value
**Infection (N = 88)[a]**	GA at delivery, d	0.01 (-0.01-0.02)	0.48		0.00 (-0.01-0.02)	0.60		0.00 (-0.01-0.01)	0.94
	Time from infection to delivery, d	0.02 (0.01-0.02)	<0.001		0.02 (0.01-0.02)	<0.001		0.02 (0.01-0.02)	<0.001
**Vaccine (N = 158)[b]**	GA at delivery, d	0.00 (-0.01-0.02)	0.69		0.01 (-0.00-0.02)	0.15		0.01 (-0.00-0.02)	0.09
	Time from 1st vaccine dose to delivery, d	0.03 (0.03-0.03)	<0.001		0.03 (0.03-0.03)	<0.001		0.03 (0.03-0.03)	<0.001

Footnote:

Linear Regression Models:
- Model 1 – Bivariate analysis of log2-transformed transfer ratio vs. variable.
- Model 2 – Multivariable analysis of log2-transformed transfer ratio vs. variable adjusting for both variables.
- Model 3 – Multivariable analysis of log2-transformed transfer ratio vs. variable adjusting for both variables and maternal obesity, hypertension and diabetes.

[a]Includes IgG-seropositive pregnant persons with symptomatic SARS-CoV-2 infection with duration between onset of symptoms or PCR positive test and delivery ≤122 days. Excludes 1 person with missing prepregnancy BMI.

[b]Includes IgG-seropositive pregnant persons with SARS-CoV-2 vaccine. Excludes 8 persons who also had history of SARS-CoV-2 infection (known PCR positive), 10 persons with missing duration between time from 1st dose of vaccine to delivery, and 1 person with missing prepregnancy BMI.

BMI, body mass index; *CI*, confidence interval; *GA*, gestational age; *IgG*, immunoglobulin G; *SARS-CoV-2*, severe acute respiratory syndrome coronavirus 2.

© 2022 Flannery DD et al. *JAMA Network Open.*
